# Analysis and Factors Associated with Measles in Larut, Matang and Selama Districts, Perak, Malaysia

**DOI:** 10.21315/mjms2020.27.5.13

**Published:** 2020-10-27

**Authors:** Asraf Ahmad Qamruddin, Reza Qamruddin, Ayu Malik

**Affiliations:** 1 Larut Matang and Selama District Health Office, Perak, Malaysia; 2 Emergency Department, Melaka Hospital, Melaka, Malaysia

**Keywords:** measles, incidence rate, immunisation, infectious diseases

## Abstract

**Objectives:**

To determine the incidence rate of measles and the factors associated with confirmed measles cases in Larut, Matang and Selama districts.

**Methods:**

Cross-sectional analysis was carried out looking at all suspected and laboratory-confirmed measles cases in Larut, Matang and Selama districts between 2015 and 2019. Multiple logistic regression analysis was used to determine the associated factors for laboratory-confirmed measles cases.

**Results:**

The incidence rate for suspected measles showed an increasing trend from 2015–2019. For laboratory-confirmed measles cases, the incidence rate showed more variation with an increase to 36.11 per million population in 2017 from 5.67 per million population in 2015. The incidence rate later decreased to 10.99 per million population in 2018 and increased again to 24.47 per million population in 2019. From multiple logistic regression analysis, cases that fulfilled the case definition of measles were more likely to be laboratory-confirmed measles. On the other hand, a prior history of measles immunisation was a protective factor.

**Conclusion:**

Measles incidence is increasing in trend. Any suspected measles cases that fulfilled the clinical case definitions need to be further investigated. Immunisation should be promoted as they are effective in preventing and eliminating measles.

## Introduction

Measles is a highly contagious disease. The disease is caused by a single-stranded ribonucleic acid (RNA) virus of the genes Morbilivirus from the Paramyxovirus family (1). The mode of transmission is by droplets from the nose, mouth or throat of an infected person to another person (2). It normally manifests as symptoms of high fever associated with cough, coryza and conjunctivitis after an average incubation period of 10–12 days from exposure (3). Several days later, this is followed by a maculopapular rash which normally starts from the face and upper neck and gradually spreading downwards.

Although the infection is typically self-limiting, some particularly serious complications can occur, particularly in children and immunocompromised individuals (4). Worldwide, in the 1980s, measles was believed to be responsible for around one to two million deaths per year, mostly in developing countries, mainly for children aged 6 years and younger (5). The estimated case fatality rate for measles is believed to be between 0.05% and 6%, with it being worse in situations of conflict (6). Of all reported cases, 18% require hospitalisation with 8%, 6% and 0.1% suffering complications such as diarrhoea, pneumonia and encephalitis, respectively. Even survivors of measles experience long-term complications. As many as 1 in 10,000 may eventually develop subacute sclerosing panencephalitis (SSPE) within 10–20 years (7). SSPE leads to neurological complications such as memory loss, and even death in late adolescence (8).

The development of the measles vaccine and its routine inclusion into childhood immunisation have significantly altered the mortality and morbidity associated with measles. There was a 75% reduction in measles-related deaths from 733,000 in the year 2000 to 146,000 in the year 2013 (9). In Malaysia, from 1982 until 2002, a single dose of measles vaccination was given to children at 9 months of age as part of the Ministry of Health Expanded Programme of Immunisation. As a result, the incidence rate of measles dropped from 65.2 cases per 100,000 of the population in 1982 to between 1.51 and 5.87 cases per 100,000 between 1989 and 1998 (10). Subsequently, from 2002, as part of the measles elimination strategy, the Ministry of Health Malaysia introduced the double-dose measles-mumps-rubella (MMR) vaccine at 12 months old and 7 years old (10). The World Health Organization (WHO) had initially set a regional target for the Western Pacific Region, of which Malaysia is a part, to eliminate measles by the year 2012 (11). Malaysia initially showed success with the incidence of measles being reduced to 2.27 cases per 100,000 individuals in 2006 and maintained at a similar level until 2010 (10). The high coverage of the measles vaccination was estimated to be around 95% in the year 2009, and, coupled with the steadily decreasing incidence of measles, it seemed that Malaysia was on track to achieve its goal of measles elimination (12). However, in 2011 and 2012, the incidence increased with outbreaks reported in a few states in Malaysia. This is believed to have been caused by the degree of population under vaccination (13). The reported incidence per 1,000,000 of the population increased from 6.6 cases in 2013 to 43.2 in 2015, 52.3 in 2017 and 59.6 in 2018 (14). In 2016, Malaysia followed the WHO recommendations and changed the measles-containing vaccine (MCV) vaccination schedule to MCV1 at 9 months of age, followed by MCV2 at 12 months of age (15). Despite this, in the year 2017 in the Larut, Matang, and Selama districts, three outbreaks of measles were reported.

The objectives of this study were to determine the incidence rate of measles in the Larut, Matang and Selama districts in Perak from 2015–2019 and to determine the factors associated with confirmed measles cases in the Larut, Matang and Selama districts between 2015 and 2019.

## Methods

A cross-sectional study was conducted between 1 January 2020 and 30 January 2020, for all cases reported as suspected measles in the online measles surveillance database (e-measles and ‘e-notifikasi’) between 1 January 2015 and 31 December 2019, in the districts of Larut, Matang and Selama in Perak. Larut, Matang and Selama are among the 11 districts in Perak with a land area of 2,046.578 km^2^ (16). The total population in 2016 was 356,200 (17).

In Malaysia, all suspected cases of measles in healthcare facilities, either government or private, are required by law to be reported under the Akta Pencegahan dan Kawalan Penyakit Berjangkit 1988 (Act 342) (18). Both ‘e-notifikasi’ and e-measles were developed and are run by the Ministry of Health, Malaysia. ‘E-notifikasi’ is an online notification system that is used by healthcare facilities to notify all notifiable disease to the District Health Office (19). E-measles was developed to standardise the reporting, investigation, and findings at the district, state, and national levels for the control and prevention of measles. All cases of measles received by the health inspector at the District Health Office from ‘e-notifikasi’ are investigated within 48 h from the time of notification. The investigation is done using an investigation form, which includes details of the patients (age, sex, date of onset of rashes and date of the specimen), past medical history of the case (immunisation history, measles immunisation status, number of doses and last measles vaccine dose date) and the measles coverage area in the locality. All of these data are then entered into the e-measles database through an online system.

Following the WHO recommendations, laboratory confirmation of measles was based on the detection of anti-measles virus IgM antibodies by enzyme-linked immunosorbent assay (ELISA) or the detection of measles virus RNA by reverse transcription-polymerase chain reaction (RT-PCR) in throat swabs, oral fluid, nasopharyngeal mucous or urine (20). All laboratory specimens for this study were sent to the National Public Health Laboratory, which is a WHO reference laboratory for measles (21). All cases of measles reported to the Larut, Matang, and Selama District Health Office between 1 January 2015 and 31 December 2019, were included. Reported cases without a laboratory or confirmatory test for measles were excluded.

The clinical case definition of measles used was a case with fever and maculopapular rash, as well as at least one of the ‘3Cs’ (cough, coryza and conjunctivitis) as advised by the Disease Control Division of the Ministry of Health, Malaysia (10).

## Data Collection and Analysis

Data collection and analysis were conducted at the Larut, Matang and Selama District Health Office. Data were downloaded from e-measles. This was followed by importing the data and analysing the data by using IBM Statistical Package for Social Science (SPSS) version 24.0.

### To Determine the Incidence Rate

The incidence rate for suspected and confirmed measles cases for the year 2015, 2016, 2017, 2018 and 2019 was calculated by using the following formula (22):

For suspected measles (per 100,000 population):

$$\frac{\text{Number of reported suspected measles in the Larut},\;\text{Matang and Selama District Health Office for the respective year}}{\text{Estimated number of population in Larut},\;\text{Matang and Selama in the year}}\times 100,000$$

For confirmed measles (per 1,000,000 population):

$$\frac{\text{Number of laboratory-confirmed measles in the Larut},\;\text{Matang and Selama District Health Office for the respective year}}{\text{Estimated number of population in Larut},\;\text{Matang and Selama in the year}}\times 1,000,000$$

Suspected measles was defined as any person diagnosed as measles by a clinician and notified to the District Health Office. Confirmed measles was defined as laboratory-confirmed cases. Estimated population in Larut, Matang and Selama for the year 2015–2019 was obtained from the record of population projection from the Department of Statistics, Malaysia.

### To Determine the Factors Associated with Confirmed Measles Cases in Larut, Matang and Selama Districts between the Year 2015 and 2019

The association between sociodemographic factors (gender, age and race), fitting the case definition of measles, immunisation factors (mumps immunisation history, dose received) and healthcare factors (distance from healthcare) was analysed using simple, and later multiple, logistic regression analysis. For the univariable analysis, simple logistic regression analysis was applied to all independent variables to determine if there was an association with laboratory-confirmed measles. The outcome of the confirmed measles was coded with binary coding: ‘0’ for cases laboratory-confirmed as non-measles and ‘1’ for cases laboratory-confirmed as measles.

Variables with a *P*-value of less than 0.25 from the univariable analysis were selected and considered for multiple logistic regression analysis. The analysis was then performed to evaluate significant factors associated with confirmed measles cases. Receiving measles immunisation was collapsed, no history of prior measles immunisation, not yet qualified for measles vaccination and unknown measles vaccination status were combined due to low cell count. Multicollinearity and interaction were checked for the final model. Fitness of the model was tested using the Hosmer-Lemeshow goodness-of-fit test, classification table and area under the receiver operating characteristics (ROC) curve in SPSS software (23). All confirmed measles cases from 2015–2019 were analysed.

The significance level for all statistical tests was set at 0.05 unless otherwise stated.

## Results

### Characteristics of the Reported Cases

Between 1 January 2015 and 31 December 2019, 359 cases of suspected measles were reported in Larut, Matang and Selama districts. Forty-seven (14.7%) of the cases were discarded as no laboratory test was performed. Table 1 shows the characteristics of the remaining 312 suspected cases. All reported cases presented with maculopapular rash and a history of fever, 133 cases (42.9%) had cough, 111 cases (34.7%) had coryza and 12 cases (3.8%) had conjunctivitis. All cases were Malaysian, except for three cases (two Chinese and one Vietnamese). There were no measles deaths reported in the Larut, Matang and Selama districts during this period. The only known complication was diarrhoea (four cases). During the 5 years, there were three measles outbreaks in the districts. All occurred in the year 2017, involving a total of seven confirmed cases.

From 312 suspected cases, 30 cases were confirmed to be measles by laboratory investigation. Majority of the cases were less than 1 year old (56.7%). Followed by more than 15 years old age group (26.7%) and the age group between 1 and 5 years old (16.7%). All confirmed cases were Malaysian citizens from Malay ethnicity, with male cases being slightly more than female cases (17 versus 13). All of them had a maculopapular rash and a history of fever. Cough, coryza or conjunctivitis (meeting the clinical case definition) was reported by 17 (56.7%) of them. From the 30 cases, 10 (33.3%) had a history of measles vaccination, 8 (26.7%) were not vaccinated and 2 (6.7%) had unknown vaccination status. Ten cases (33.3%) were not yet qualified for measles vaccination according to the Malaysia National Immunisation Programme. Only 1 case (3.3%) had documented two doses of measles vaccination, 8 cases (26.7%) had one dose of measles vaccination, 18 cases (60.0%) had not received any and 3 cases (10.0%) had an unknown number of measles vaccination doses received.

### The Incidence Rate for Suspected Measles and Confirmed Measles

**Figure 1:**
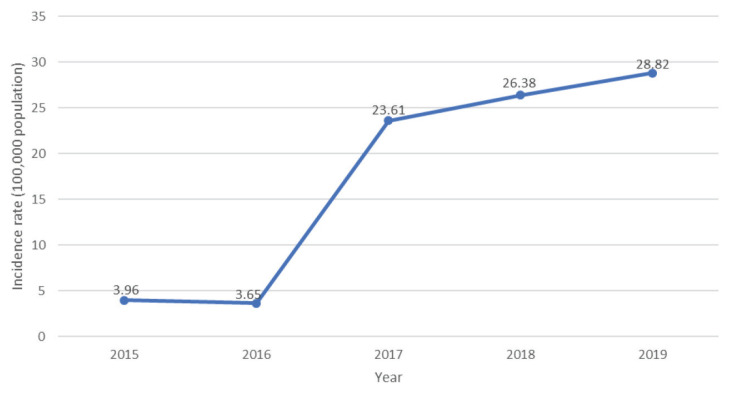
Incidence rate of suspected measles cases reported in Larut, Matang and Selama District Health Office between the year 2015 and 2019

**Figure 2:**
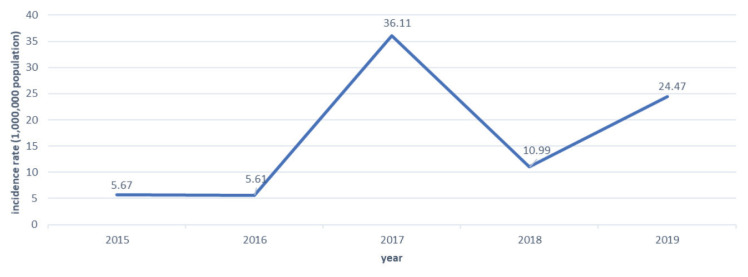
Incidence rate of confirmed measles cases in Larut, Matang and Selama districts between the year 2015 and 2019

Figure 1 shows the incidence rate of reported suspected measles cases per 100,000 of the population yearly between 2015 and 2019 in the Larut, Matang and Selama districts. The incidence rate of confirmed measles cases per million of the population from year 2015–2019 is shown in Figure 2.

### Factors Associated with Confirmed Measles Cases

Table 2 shows the simple and multiple logistic regression analyses of factors associated with confirmed measles cases in the districts of Larut, Matang and Selama districts during the study period among the reported suspected measles cases.

From the multiple logistic regression analysis, only cases that fulfilled the case definition and had a history of receiving measles vaccination were significantly associated with confirmed measles cases after adjusting for other factors. The Hosmer-Lemeshow goodness-of-fit test showed the model was fit with a *P*-value of 0.933. The overall percentage of the classification table was 91% and the area under the ROC curve was 0.846 (95% CI: 0.784, 0.908).

## Discussion

### Characteristics of the Cases

Most of the suspected and confirmed measles cases were among those under 1 year old, followed by the 1- to 5-year-old age group. Historically, infants were believed to be less affected by measles; however, this may no longer be true (24). Of the 17 confirmed cases under 1 year old, four cases were below the age of 9 months. Therefore, not qualified for the first dose of MCV vaccination in Malaysia. Thirteen of the other cases were at or above the age for the first dose of MCV vaccinations. It has long been believed that infants under 1 year old were protected by the anti-measles antibodies transmitted from their mothers during pregnancy which lasted until the end of their first year (25). However, this was shown to be partly wrong with documented cases of measles occurring in infants under 1 year old and sometimes younger than 6 months (26). A systematic review of studies on measles maternal antibodies in infants in measles elimination settings reported that despite around 80%–100% of infants being protected from measles at birth, there is limited protection in infants older than 4 months old (27).

In Malaysia, the first dose of MCV is given at 9 months as recommended by the WHO. Therefore, the questions of whether WHO should recommend and whether Malaysia should implement earlier age for the first dose of MCV vaccination need to be answered. If the first dose is administered too early, the immune response can be blunted due to immunologic immaturity and interference of the maternal antibodies (28). Vaccination before the age of 6 months often fails to induce seroconversion due to the immaturity of the infant’s immune system as well as the presence of neutralising maternal antibodies (29). Even at the age of 9 months, primary vaccination failures could occur in up to 10%–15% of infants (30). Therefore, the recommended age for vaccination must be balanced between the risk of primary vaccine failure, which decreases with increasing age, with the risk of measles virus infection occurring before vaccination, which increases with age. The WHO currently recommends two doses of MCV vaccination.

Studies have shown that in children who did not respond to the first dose of the measles vaccine, almost 95% developed protective immunity after the second dose (31). This is echoed by the WHO position paper on the Measles Vaccine, which advises MCV1 vaccination for countries with ongoing transmission of measles should be at 9 months of age and the MCV2 dose should be administered with a minimum interval between MCV1 and MCV2 of 4 weeks (31). However, the WHO advises a supplementary dose of MCV should be given to infants from 6 months of age in the following situations: i) during a measles outbreak as part of intensified service delivery; ii) during campaigns in settings where the risk of measles among infants < 9 months of age remains high; iii) internally displaced populations and refugees and populations in conflict zones; iv) individual infants at high risk of contracting measles; v) infants travelling to countries experiencing measles outbreaks; and vi) infants known to be HIV-infected or exposed (born to an HIV-infected woman).

There were slightly more males than females in the confirmed cases (17 versus 13). This is most likely due to the higher number of reported suspected measles cases among males compared to females. An analysis using Pearson Chi-square analysis found that there is no significant difference among genders (*P* = 0.610).

Malay ethnicity was reported as the highest number of suspected measles cases. Therefore, unsurprisingly, all of the confirmed measles cases were from this ethnicity. Although Malay is the major ethnic group in Larut, Matang and Selama districts at almost 65% of the population, this does not explain the disproportionate incidence rate among the ethnic group (17). Records of refused vaccination kept at the district level from 2015–2019 showed a total of 212 children were not vaccinated for MMR due to parental refusal. The two most common reasons for refusal given were that they were worried about the safety of the vaccine and religious excuses. All cases of documented refusals were of Malay ethnicity. Most of the cases were close to health facilities, with the mean distance to the nearest healthcare facilities being less than 5 km. A study has shown that a distance of more than 20 km significantly affects the prognosis of measles cases (32).

### The Incidence Rate for Reported and Confirmed Measles

In 2016, there was a slight decrease in the incidence rate of suspected measles cases in the districts. Followed by increasing in the trend of suspected measles cases every year. An almost eight-fold increase in the incidence rate in 2017 was seen compared to 2016. Further, the suspected measles incidence rate was at an all-time high (28.82 per 100,000 individuals) in 2019. Compared to the incidence rate of suspected measles for the Perak state itself, a similar trend was observed when the incidence rate took a slight dip in 2016 from 7.63 to 7.16 per 100,000 of the population and then showed an increasing trend but not as drastically as seen in Larut, Matang and Selama districts to 16.68 in 2017, 24.36 in 2018 and 19.62 per 100,000 of the population in 2019.

For the laboratory-confirmed measles cases, the trend showed a similar slight decline in 2016, followed by drastic increases in 2017. Most likely, the increase in both the suspected and confirmed measles cases in 2017 occurred as a result of three outbreaks that occurred in the Larut, Matang and Selama District Health Office during this period of time. All three outbreaks occurred in June involving three cases, two cases and three cases. Epidemiological investigation revealed all three outbreaks had an epidemiological link with cases from outside the state, which could have been the source of infection. Outbreaks were also reported in other parts of Perak, leading to increases in reported and suspected measles incidence rates for the Perak state as well.

The relatively low incidence rate of both reported and suspected measles cases before 2017 in the districts of Larut, Matang and Selama could also be due to underreporting of cases. Therefore, it might not fully reflect the burden of the disease. Studies have shown that physicians tend to underdiagnose and underreport mandatory reporting diseases when considered not severe (33, 34). The 2017 outbreaks increased physicians’ awareness and led to physicians having a higher index of suspicion for measles and increased notifications of suspected measles cases despite the incidence rates in 2018 and 2019 for confirmed measles showed a decline (35).

### Factors Associated with Confirmed Measles

Four main factors were included in the analysis, namely sociodemographic factors (age, gender and ethnicity), fulfilling the case definition, immunisation status and the distance from the house to the nearest facility providing vaccination services. After adjusting for other factors, two factors were significantly associated with laboratory-confirmed measles in the study group. The two factors were fulfilling the case definition and receiving measles immunisation.

Cases that fulfilled the clinical case definitions (i.e. cases that had a fever, rash and one of the ‘3Cs’ (cough, coryza or conjunctivitis) had 6.72 times higher odds of being true measles when other factors were considered. Studies have shown that the clinical case definition has high predictive values in diagnosing measles. A study evaluating the measles clinical case definition in New York City reported the negative predictive value of the case definition at 98% (36). Similarly, Sarmiento et al. (37) reported a negative predictive value of 86% of the clinical case definitions in their study in Venezuela. Furthermore, a review of four studies reported that the sensitivity of the clinical case definition was high (76%–88%). Therefore, WHO, the Centre for Disease Control and Prevention and Ministry of Health Malaysia have agreed on the similar clinical case definitions, as most measles cases will fulfil this definition.

Immunisation status significantly protected against confirmed measles in the study group. After adjusting for other factors, cases who never received MCV, were not yet qualified for MCV vaccination or had unknown vaccination status had 1.72 times higher odds of being confirmed measles compared to those who did receive the vaccination. A systematic review of 138 studies found that a single MCV vaccine reduces the risk of measles infection by 95% and 96% for two doses (38). Vaccination has dramatically reduced measles in all countries of the world. In the United States, after the MCV vaccination was introduced in the 1970s, the incidence rate dropped by more than 95% (36). For infants who receive one dose of MCV at 8–9 months of age, 89.6% of them seroconvert (39). Having two doses of MCV increases the proportion to almost 100%. Despite being highly contagious, measles has all of the components of an eradicable disease: there is a safe and highly effective vaccine, it has a readily diagnosable clinical syndrome and there is no animal reservoir (40). Measles vaccination has proven to be effective at not only preventing infection in individuals but also in the community through herd immunity. Therefore, maintaining at least 95% of the population as immune will ensure herd immunity and maintain contact immunity in the population to effectively stop the virus from spreading from person to person (41, 42, 43).

Since the study population consisted of the population in the Larut, Matang and Selama districts, the result cannot be generalised to other populations. We had no access to external information other than the online notification database. Therefore, the incidence of suspected and confirmed measles depended on the notification by physicians. The projected population provided by the local department of statistics was used to calculate the population in Larut, Matang and Selama districts from 2015 to 2019. Therefore, the population used is not the true population but rather an estimation.

The strength of this study was that the confirmatory test for measles was done in a single laboratory, which is a WHO-recognised laboratory for measles, thereby reducing interlaboratory bias. We also used the two online databases for measles surveillance in Malaysia. Around 87% of the suspected cases in the online databases during the study duration were laboratory-confirmed cases, which are above the WHO recommended standard ≥ 80% (31).

## Conclusion

From this study, suspected and confirmed measles incidence rates in the districts showed a generally increasing trend. Cases that fulfilled the clinical case definitions need to be investigated thoroughly, as they are more likely to be true cases of measles. Immunisation is an effective method for preventing measles and should be promoted to eliminate measles.

## Figures and Tables

**Table 1:** Characteristics of the reported measles cases in Larut, Matang and Selama District Health Office from 1 January 2015–31 December 2019 (*n* = 312)

Variable	*n* (%)
**Sociodemographic**	
**Age (years)**	
< 1 year	149 (47.8)
1–5 years	98 (31.4)
6–14 years	33 (10.6)
> 15 years	32 (10.2)
Gender	
Male	163 (52.2)
Female	149 (47.8)
Ethnicity	
Malay	281 (90.1)
Chinese	23 (7.4)
Indian	5 (1.5)
Others	3 (1.0)
Fulfil case definition	
Yes	174 (55.8)
No	138 (44.2)
**Immunisation**	
Received measles immunisation	
Yes	162 (51.9)
No	139 (44.6)
Not known	11 (3.5)
**Dose received**	
0	139 (46.6)
1	67 (20.6)
2	27 (8.4)
Not known	79 (24.4)
**Healthcare**	
Distance from house to healthcare (km)	4.4 (2.18)^a^

Note:

a mean (SD)

**Table 2:** Simple and multiple logistic regression analysis of factors for confirmed measles cases in Larut, Matang and Selama District Health Office between the year 2015 and 2019

Variable	Wald statistic	df	Crude OR (95% CI)	*P*-value	Adj. OR (95% CI)	*P-*value
**Sociodemographics**						
Age (years)	8.953	3	1			
< 1 year			0.42 (0.14, 1.17)	0.097		
1–5 years			0.01 (0.01, 0.01)	0.998		
6–14 years			2.59 (1.01, 6.67)	0.049		
> 15 years						
Gender	0.517	1	1			
Male			0.75 (0.35, 1.63)	0.998		
Female						
Ethnicity	< 0.001	1	1			
Malay			0.01 (0.01, 0.01)	0.073		
Others						
**Clinical symptoms**						
Fulfill case definition	9.950	1	5.70 (1.93, 16.80)	0.002*	6.72 (2.12, 21.28)	0.001*
**Immunisation**						
Received measles immunisation						
Yes	0.722	1	1		1	0.014*
Others			1.72 (1.04, 6.75)	0.026*	1.98 (1.42, 8.83)	
Dose received						
0	2.752	3	1			
1			1.67 (0.73, 3.80)			
2			0.37 (0.05, 2.87)			
Not known			0.01 (0.01, 0.01)			
**Healthcare**						
Distance from house to healthcare (km)	0.076	1	0.98 (0.84, 1.14)	0.783		

Notes: Constant –2.751; Forward LR and Manual method applied; No multicollinearity and no interaction detected; Hosmer-Lemeshow test *P*-value = 0.933; Classification table 91% correctly classified; Area under receiver operating characteristic (ROC) was 84.5;

* *P* < 0.05 ;

df = degree of freedom; OR = odds ratio; CI = confidence interval
